# Epidermal Growth Factor Receptor Tyrosine Kinase Inhibitor Erlotinib Induces Dry Skin via Decreased in Aquaporin-3 Expression

**DOI:** 10.3390/biom10040545

**Published:** 2020-04-03

**Authors:** Nobutomo Ikarashi, Miho Kaneko, Tomofumi Watanabe, Risako Kon, Makana Yoshino, Takatoshi Yokoyama, Riho Tanaka, Naoya Takayama, Hiroyasu Sakai, Junzo Kamei

**Affiliations:** Department of Biomolecular Pharmacology, Hoshi University, 2-4-41 Ebara, Shinagawa-ku, Tokyo 142-8501, Japan; miho.k-611@ezweb.ne.jp (M.K.); rvimdkamagk@gmail.com (T.W.); r-kon@hoshi.ac.jp (R.K.); macaron0921@gmail.com (M.Y.); boc-2153-m.f@i.softbank.jp (T.Y.); s141155@hoshi.ac.jp (R.T.); s151150@hoshi.ac.jp (N.T.); sakai@hoshi.ac.jp (H.S.); kamei@hoshi.ac.jp (J.K.)

**Keywords:** aquaporin-3, dry skin, erlotinib, EGFR, ERK

## Abstract

An adverse reaction of dry skin occurs frequently during treatment with anticancer epidermal growth factor receptor tyrosine kinase inhibitors (EGFR-TKIs). In this study, we conducted basic research to clarify the mechanism of EGFR-TKI-induced dry skin and propose new treatments or preventative measures. Dermal water content was significantly lower in the erlotinib-treated mice than in the control group. An assessment of the expression levels of functional genes in the skin revealed that only the expression of the water channel aquaporin-3 (AQP3) was significantly decreased in the erlotinib-treated group. When erlotinib was added to epidermal keratinocyte HaCaT cells, the expression levels of both AQP3 mRNA and protein decreased. Erlotinib treatment also significantly decreased the expression levels of phospho-EGFR and phospho-extracellular signal-regulated kinase (ERK), both in HaCaT cells and mouse skin. Dry skin due to erlotinib may be caused by the decreased expression of AQP3 in the skin, thereby limiting water transport from the vascular side to the corneum side. The decrease in AQP3 may also be attributable to ERK suppression via inhibition of EGFR activity by erlotinib. Therefore, substances that increase AQP3 expression may be effective for erlotinib-induced dry skin.

## 1. Introduction

Epidermal growth factor receptors (EGFRs) are intrinsic tyrosine kinase receptors expressed in the cell membranes of normal tissues in the skin, lungs, gastrointestinal tract, etc. EGFRs recognize epidermal growth factors (EGFs) and control cell growth and development. EGFRs are also overexpressed in various cancer cells [1], indicating their involvement in cancer cell growth, apoptosis survival, neoangiogenesis, and metastasis [2]. For this reason, the EGFR is a target molecule for inhibiting cancer cell growth, and various EGFR tyrosine kinase inhibitors (EGFR-TKIs) have been developed for proactive use against refractory cancers [3,4,5]. However, since EGFR-TKIs act not only on cancer cells but also on normal cells, a variety of adverse reactions may occur.

Adverse reactions of EGFR-TKIs frequently occur on the skin [6,7,8]. For example, many patients complain of an acneiform rash or dry skin during erlotinib treatment. Acneiform rash is considered to be caused by increased sebum secretion and associated skin inflammation [9]. Currently, topical steroids are used for treatment, and the anti-inflammatory effects of these drugs can relieve the symptoms [10]. Although skin dryness is suspected to be attributed to the toxicity of erlotinib to skin [11], the detailed mechanism remains unknown, and symptomatic therapy using a moisturizer is used for dry skin. However, a complete cure has still not been obtained. Erlotinib administration is sometimes discontinued due to dry skin. Therefore, suppression of dry skin can maximize the efficacy of erlotinib in cancer treatment.

As discussed above, EGFR-TKI-induced dry skin not only causes a decrease in patient quality of life, but also influences the continuation of cancer treatment. Therefore, we must aim to understand the cause of EGFR-TKI-induced dry skin and provide an appropriate treatment. In this study, we conduct basic research using erlotinib to clarify the mechanism of dry skin caused by EGFR-TKIs and propose a new treatment or preventative measure.

## 2. Materials and Methods

### 2.1. Materials

Erlotinib was purchased from LC Laboratories (Woburn, MA, USA). Gefitinib was purchased from FUJIFILM Wako Pure Chemical Corporation (Osaka, Japan). TRI reagent was purchased from Sigma-Aldrich Corp. (St. Louis, MO, USA). A high-capacity cDNA synthesis kit was purchased from Applied Biosystems (Foster City, CA, USA). The rabbit anti-rat aquaporin-3 (AQP3) antibody was purchased from Alomone Labs (Jerusalem, Israel). The mouse anti-rabbit glyceraldehyde-3-phosphate dehydrogenase (GAPDH) antibody and sheep anti-mouse IgG-HRP antibody were purchased from Merck Millipore (Darmstadt, Germany). The donkey anti-rabbit IgG-HRP antibody was purchased from Santa Cruz Biotechnology Inc. (Santa Cruz, CA, USA). The rabbit anti-human EGFR antibody, rabbit anti-human phospho (p)-EGFR antibody, rabbit anti-human p44/42 mitogen-activated protein kinase (MAPK) antibody, and rabbit anti-human p-p44/42 MAPK antibody were purchased from Cell Signaling Technology (Beverly, MA, USA). Alexa Fluor 488 donkey anti-rabbit IgG was purchased from Thermo Fisher Scientific (Waltham, MA, USA). An enhanced chemiluminescence (ECL) prime detection reagent was purchased from GE Healthcare (Chicago, IL, USA). The cell proliferation reagent water-soluble tetrazolium salt (WST-1) was purchased from Roche (Mannheim, Germany).

### 2.2. Animals and Treatments

Male HR-1 hairless mice (seven weeks old) were purchased from Japan SLC, Inc. (Shizuoka, Japan). The mice were housed at 24 ± 1 °C and 55 ± 5% humidity with 12 h of light (08:00–20:00). The study was conducted upon approval (approval No. 27–130, Approval date: 29/3/2016) in accordance with the Hoshi University Guiding Principles for the Care and Use of Laboratory Animals.

Erlotinib (50 mg/kg/day) [12] or 1% polyoxyethylene sorbitan monooleate solution was administered orally to the mice for 14 days. Before autopsy, body weight, food intake, water intake, and urine volume were measured using metabolic cages. The dermal water content was measured using a Corneometer CM825 (Courage + Khazaka, Cologne, Germany). Transepidermal water loss was measured using a Tewameter TM300 (Courage + Khazaka, Cologne, Germany). After the end of the treatment period, the mice were anesthetized, and the liver, kidney, and skin were removed.

### 2.3. HaCaT Cell Culture

HaCaT cells (Cell Line Service, Eppelheim, Germany) were cultured using Dulbecco’s modified Eagle medium (DMEM, 100 U/mL penicillin G potassium, 100 μg/mL streptomycin, and 10% fetal bovine serum). HaCaT cells were plated in a 24-well plate, 96-well plate, or 10-cm dish at a cell density of 6 × 10^4^ cells/cm^2^ and incubated for 2 days. Then, DMSO (final concentration; 0.1%), erlotinib (0.125–8 μM), or gefitinib (0.125–2 μM) was added, and cells were incubated for 24 h.

### 2.4. Real-Time RT-PCR

RNA was extracted from mouse tissue samples or HaCaT cells using TRI reagent. A high-capacity cDNA synthesis kit was used to synthesize cDNA from 1 μg of RNA. Target gene expression was analyzed by real-time RT-PCR (CFX Connect Real-Time System, Bio-Rad Laboratories, Hercules, CA, USA) using the primers listed in Table 1. mRNA gene expression levels were normalized to m18S rRNA or hRPL30 gene expression levels.

### 2.5. Hematoxylin and Eosin (H&E) Staining

Skin samples isolated from mice were immersed in 10% neutral buffered formalin for tissue fixation. The tissue samples were embedded in paraffin and cut into 3-μm sections mounted on glass slides. The slides were deparaffinized and stained with hematoxylin followed by eosin. The slides were dehydrated in alcohol, cleared in xylene, and covered for microscopic examination. The slides were evaluated by a pathologist in a blinded manner, and skin damage was assessed.

### 2.6. Electrophoresis and Western Blotting

Mouse skin or HaCaT cells were processed by a previous method [13,14] and used for Western blotting. Proteins were separated using sodium dodecyl sulfate-polyacrylamide gel electrophoresis and then transferred to a polyvinylidene difluoride membrane. The proteins in the membrane were probed with primary and secondary antibodies. The recognized proteins were detected using an ECL prime detection reagent. The protein immunocomplexes were visualized using a Lumino Image Analyzer (ImageQuant LAS500 system, GE Healthcare, Chicago, IL, USA).

### 2.7. Immunohistochemistry

The mouse skin or HaCaT cells were postfixed in 4% paraformaldehyde. The tissues were embedded, and the frozen blocks were sectioned into 10-μm slices on glass slides. The sections were reacted with a rabbit anti-rat AQP3 antibody. The sections were treated with an Alexa Fluor 488 donkey anti-rabbit IgG antibody, and the slides were covered and observed under a fluorescence microscope.

### 2.8. Cell Viability

HaCaT cells were plated and incubated for 48 h after the addition of erlotinib. After each well was washed, WST-1 was added at a ratio of 10/100 µL medium, and cells were incubated for 2 h at 37 °C in a CO_2_ incubator. The absorbance at 450 nm/620 nm was measured using a microplate reader.

### 2.9. Statistical Analysis

Numerical data are expressed as the mean ± standard deviation (SD). The significance of the differences was examined using Dunnett’s test and Student’s t-test.

## 3. Results

### 3.1. Dermal Water Content, Transepidermal Water Loss, and Skin Inflammation Findings

The dermal water content and transepidermal water loss in the mice were measured on day 14 of erlotinib administration.

**Figure 1:**
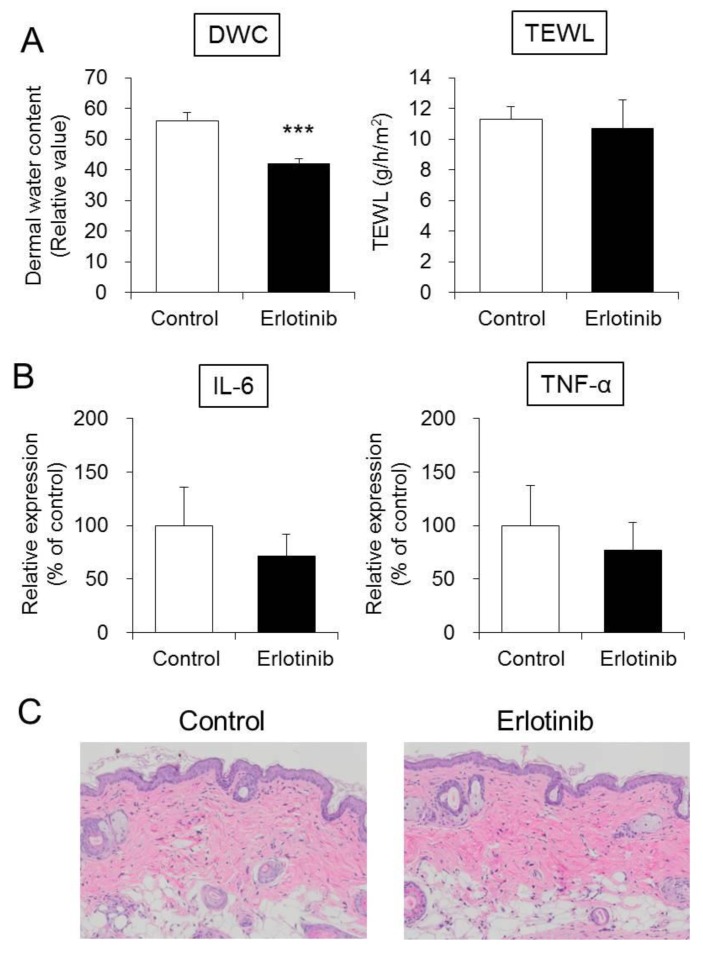
Dermal water content (DWC), transepidermal water loss (TEWL), and skin inflammation findings. Erlotinib was administered orally to the mice for 14 days, and the DWC and TEWL were measured (**A**). IL-6 and TNF-α mRNA expression levels in the skin were measured by real-time RT-PCR. After normalization to 18S rRNA, the data are presented with the mean value of the control group set at 100% (mean ± SD, *n* = 5; *** *p* < 0.001) (**B**). Skin tissue was assessed by H&E staining (**C**).

A significant decrease in the dermal water content by approximately 25% was noted in the erlotinib group compared with the control group. No difference in transepidermal water loss was noted between the two groups (Figure 1A). Skin lesions were examined by hematoxylin and eosin (H&E) staining, and no abnormalities were observed (Figure 1C). The skin findings in the erlotinib group were similar to those in the control group. Furthermore, the mRNA expression levels of interleukin-6 (IL-6) and tumor necrosis factor- α (TNF-α) in the skin of the erlotinib-treated group did not differ from those of the control group (Figure 1B).

These results demonstrate that 14 days of erlotinib administration cause dry skin in mice without affecting skin barrier function or causing apparent skin tissue damage.

### 3.2. Expression Levels of Functional Genes in Mouse Skin

Dermal water content is maintained by various factors, including filaggrin, loricrin, ceramides, hyaluronic acid, and collagen. A disruption in the balance between these factors is known to lead to a decrease in dermal water content [15,16,17]. Thus, we analyzed the expression levels of functional genes in the skin to investigate the mechanism of erlotinib-induced dry skin.

**Figure 2:**
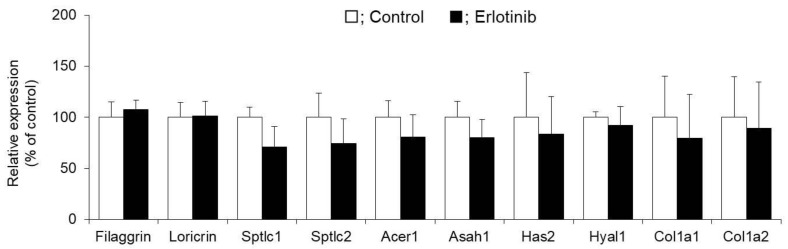
Expression levels of functional genes in mouse skin. Erlotinib was administered orally to the mice for 14 days, and the filaggrin, loricrin, Sptlc1, Sptlc2, Acer1, Asah1, Has2, Hyal1, Col1a1, and Col1a2 mRNA expression levels in the skin were measured by real-time RT-PCR. After normalization to 18S rRNA, the data are presented with the mean value of the control group set at 100% (mean ± SD, *n* = 5).

No difference was observed in the mRNA expression levels of filaggrin and loricrin in the skin between the erlotinib-treated group and the control group. In addition, there was also no difference in the mRNA expression levels of ceramide synthase (Sptlc1 and Sptlc2), ceramidase (Acer1 and Asah1), hyaluronan synthase (Has2), hyaluronidase (Hyal1), or collagens (Col1a1 and Col1a2) between the two groups (Figure 2).

Based on the above results, these genes are unlikely to be involved in skin dryness after erlotinib administration.

### 3.3. Expression Levels of AQP in Mouse Skin

AQPs act as selective channels allowing water transport. In humans, 13 types of AQPs, numbered from AQP0 to AQP12, are expressed in various tissues [18]. Many AQP family members also exist in the skin and play important roles in maintaining skin moisture and skin function. To investigate the mechanism of erlotinib-induced dry skin, we conducted an analysis on the expression of AQPs.

**Figure 3:**
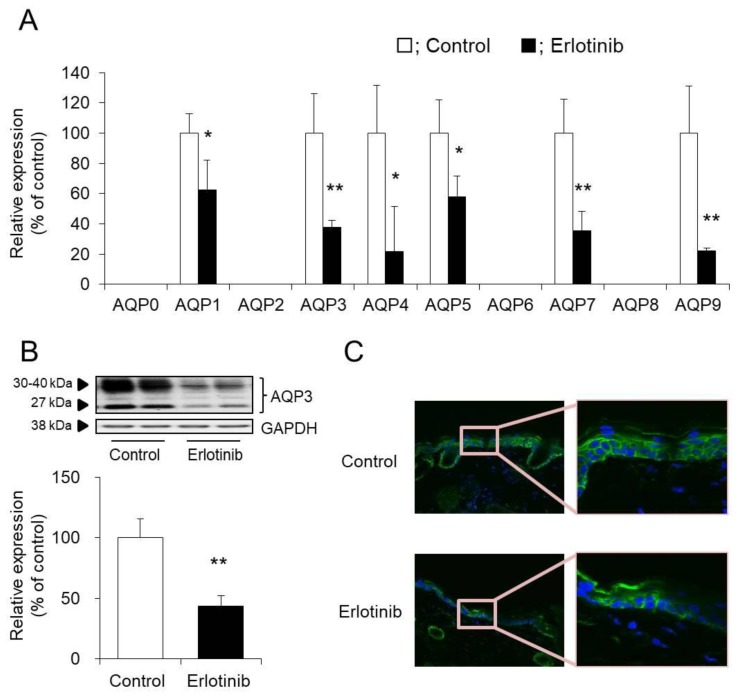
Expression levels and distribution of aquaporin (AQP) in mouse skin. Erlotinib was administered orally to the mice for 14 days, and the AQP (AQP0-9) mRNA expression level in the skin was measured by real-time RT-PCR. After normalization to 18S rRNA, the data are presented with the mean value of the control group set at 100% (**A**). The protein expression of AQP3 in the skin was analyzed by Western blotting. After normalization to glyceraldehyde-3-phosphate dehydrogenase (GAPDH), the data are shown with the mean value of the control group set at 100% (mean ± SD, *n* = 5; * *p* < 0.05 and ** *p* < 0.01) (**B**). AQP3 (green) and nuclei (blue) in mouse skin were immunostained (**C**).

Of the analyzed AQP family members, AQP1, AQP3, AQP4, AQP5, AQP7, and AQP9 were confirmed in the skin. Furthermore, the mRNA expression levels of all of these AQPs were found to be lower in the erlotinib group than in the control group (Figure 3A).

AQP3, which is the AQP most abundantly expressed in the skin, was analyzed by Western blotting. The signal bands of AQP3 were detected at approximately 27 kDa and 30–40 kDa, which reportedly correspond to AQP3 without glycosylation (27 kDa) and with glycosylation (30–40 kDa), respectively [19,20]. The presence or absence of glycosylation is known to impact AQP stability and migration into the cell membrane but has no effect on water penetrability [21,22,23]. Therefore, in this study, the sum of these bands was analyzed as the expression level of AQP3.

AQP3 protein expression in the skin was found significantly reduced in the erlotinib-treated group to approximately 50% of that in the control group (Figure 3B). Immunohistochemical staining revealed decreased AQP3 expression in the entire epidermis after the administration of erlotinib (Figure 3C).

Based on the above findings, decreased AQP3 expression may be involved in dry skin after the administration of erlotinib.

### 3.4. Effect of Erlotinib on AQP3 Expression in HaCaT Cells

We added various concentrations of erlotinib to HaCaT cells, a line of human epidermal keratinocytes, and analyzed the expression levels of AQP3 mRNA and protein.

**Figure 4:**
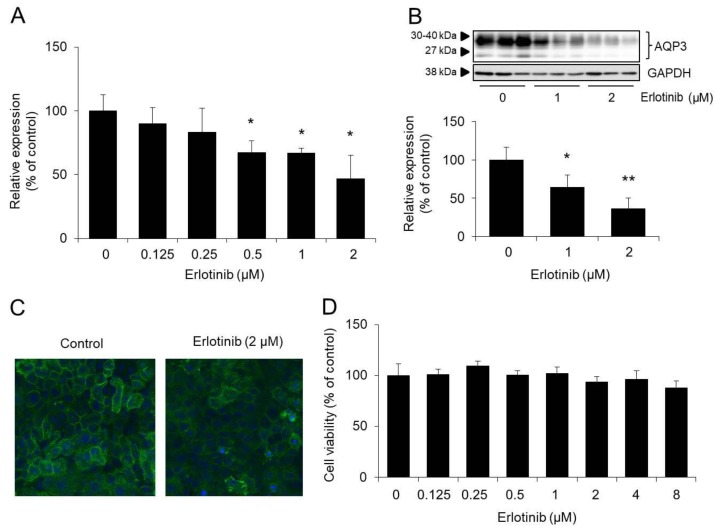
Effect of erlotinib on AQP3 expression in HaCaT cells. Erlotinib was added to HaCaT cells, and the cells were incubated for 24 h. The AQP3 mRNA expression level was measured by real-time RT-PCR. After normalization to RPL30, the data are presented with the mean value of the control group set at 100% (**A**). The protein expression of AQP3 in HaCaT cells was analyzed by Western blotting. After normalization to GAPDH, the data are shown with the mean value of the control group set at 100% (**B**). AQP3 (green) and nuclei (blue) in HaCaT cells were immunostained (**C**). Cell viability was analyzed by the reagent water-soluble tetrazolium salt (WST-1) assay (**D**) (mean ± SD, *n* = 5; * *p* < 0.05 and ** *p* < 0.01).

The expression level of AQP3 mRNA at 24 h after the addition of erlotinib decreased in a concentration-dependent manner, with erlotinib concentrations of 0.5 μM, 1 μM, and 2 μM resulting in significantly lower levels than in the control group (Figure 4A). This decrease in AQP3 expression was also observed with the protein (Figure 4B). Immunohistochemical staining confirmed decreased migration of AQP3 to the cell membrane after the addition of erlotinib (Figure 4C). Based on the results of a WST-1 assay, the decrease in AQP3 after erlotinib treatment was unlikely to be caused by cytotoxicity (Figure 4D).

The above findings suggest that erlotinib acts directly on epidermal keratinocytes and decreases the expression of AQP3.

### 3.5. Effect of Erlotinib on the Phosphorylation of EGFR and ERK in HaCaT Cells

Erlotinib inhibits the growth of cancer cells and displays anticancer activity by selectively inhibiting tyrosine kinase autophosphorylation of the EGFR of cancer cells and suppressing the activity of its downstream Ras/MAPK signaling pathway [24]. Recently, the Ras/MAPK pathway has also been demonstrated to be involved in the expression regulation mechanism of AQP3 [25]. Thus, the mechanism by which erlotinib decreases AQP3 expression was analyzed by focusing on the Ras/MAPK pathway.

**Figure 5:**
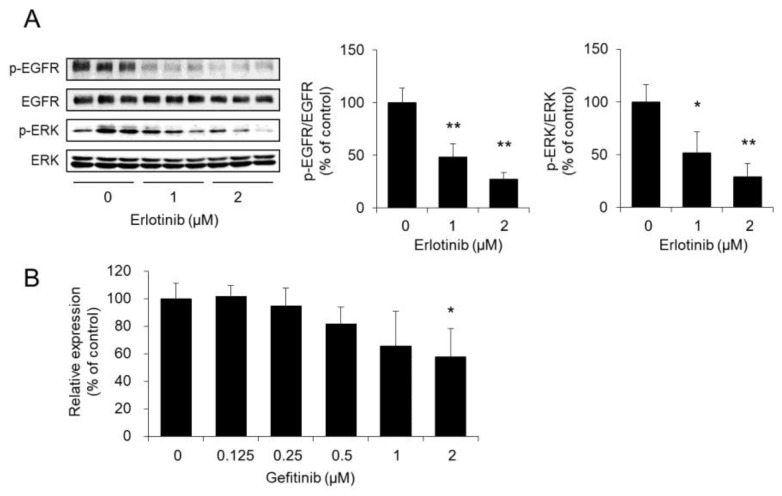
Effect of erlotinib on the phosphorylation of epidermal growth factor receptors (EGFRs) and extracellular signal-regulated kinase (ERK) in HaCaT cells. Erlotinib was added to HaCaT cells, and the cells were incubated for 24 h. The protein expression of phospho (p)-EGFR and p-ERK in HaCaT cells was analyzed by Western blotting. After normalization to EGFR and ERK, the data are shown with the mean value of the control group set at 100% (**A**). Gefitinib was added to HaCaT cells, and the cells were incubated for 24 h. The AQP3 mRNA expression level was measured by real-time RT-PCR. After normalization to RPL30, the data are presented with the mean value of the control group set at 100% (**B**) (mean ± SD, *n* = 5; * *p* < 0.05 and ** *p* < 0.01).

The level of p-EGFR/EGFR in erlotinib-treated HaCaT cells compared with that in control cells decreased significantly in a concentration-dependent manner. In addition, the level of p-ERK/ERK also decreased significantly with erlotinib treatment in a concentration-dependent manner (Figure 5A).

When the EGFR-TKI gefitinib was added to HaCaT cells in the same manner as erlotinib, the expression level of AQP3 mRNA decreased significantly (Figure 5B).

These results suggest that erlotinib may suppress the phosphorylation of EGFR and ERK and decrease AQP3 expression in HaCaT cells.

### 3.6. Effect of Erlotinib on the Phosphorylation of EGFR and ERK in Mouse Skin

Erlotinib inhibits EGFR and suppresses the activity of the Ras/MAPK pathway in HaCaT cells. Thus, we investigated whether the phosphorylation of EGFR and ERK was similarly suppressed in the skin of an erlotinib-induced dry skin mouse model.

**Figure 6:**
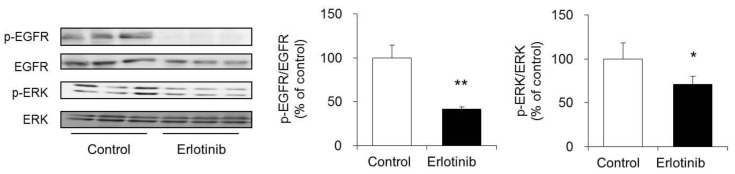
Effect of erlotinib on the phosphorylation of EGFR and ERK in mouse skin. Erlotinib was administered orally to the mice for 14 days, and the protein expression of p-EGFR and p-ERK in the skin was analyzed by Western blotting. After normalization to EGFR and ERK, the data are shown with the mean value of the control group set at 100% (mean ± SD, *n* = 5; * *p* < 0.05 and ** *p* < 0.01).

In the skin of mice treated with erlotinib, p-EGFR/EGFR levels were significantly lower than those in the control group. Furthermore, the level of p-ERK/ERK was also significantly lower in the erlotinib group than in the control group (Figure 6).

These results suggest that EGFR and ERK phosphorylation is also suppressed in the skin of an erlotinib-induced dry skin mouse model.

## 4. Discussion

In this study, we conducted basic research to clarify the mechanism of erlotinib-induced dry skin and to propose new treatment strategies or preventative measures. Erlotinib administration to mice caused no changes in liver or kidney weights. Water intake, food intake, urine volume, and body weight were also similar between the erlotinib and control groups. No marked toxicity was observed (data not shown). An examination of skin condition revealed no apparent tissue damage or inflammatory reaction and maintained barrier function (Figure 1). However, dermal water content was significantly lower in the erlotinib group than in the control group, and dry skin was also observed (Figure 1A). This result was similar to the clinically observed skin condition [6,7,8]. In conclusion, the 14 day oral administration of erlotinib to mice at a dose of 50 mg/kg produced a model that reflects the clinically observed dry skin.

We focused on the factors involved in skin moisturizing and analyzed the mechanism of erlotinib-induced dry skin using this model mouse. No differences were observed in the expression levels of filaggrin, loricrin, ceramide synthase, ceramidase, hyaluronan synthase, hyaluronidase, or type I collagen between the erlotinib and control groups (Figure 2). In contrast, both the mRNA and protein expression of the water channel protein AQP3 were significantly lower in the erlotinib group than in the control group (Figure 3). AQP3 in the skin is believed to be involved in water transport from the vascular side to the corneum side. Decreased dermal water content has been reported in AQP3 knockout mice [26,27,28]. Furthermore, skin AQP3 expression has been shown to be reduced in the presence of psoriasis [29], leukoplakia [30,31], diabetes mellitus [32], and aging [33,34], each of which cause dry skin. In this manner, AQP3 in the skin plays a crucial role in skin moisture retention. Considering the above, erlotinib-induced dry skin may be attributable to the limited water transport from the vascular side to the corneum side caused by decreased AQP3 expression. AQP3 transports both glycerol and water, controls glycerol content in the epidermis, and thus regulates skin hydration [35,36]. In this study, although glycerol levels in the skin were not measured, it was considered that glycerol levels were decreased in erlotinib-treated mice. In mammals, 13 types of AQPs numbered from AQP0 to AQP12 are expressed. An analysis of the mRNA expression levels of these AQP family members revealed the expression of AQP1, AQP4, AQP5, AQP7, and AQP9 in mouse skin, in addition to AQP3. All of these expression levels were decreased significantly by the administration of erlotinib (Figure 3A). The functions of these AQP family members in the skin are poorly understood, and their expression levels were significantly lower than that of AQP3; therefore, we believe that they are unlikely to be involved in erlotinib-induced dry skin.

Erlotinib decreased the level of AQP3 in the skin. To date, various inflammatory cytokines are known to decrease the expression of AQPs. For example, TNF-α and IL-6 reportedly decrease the expression of AQP3 in epidermal keratinocytes [37,38]. Although erlotinib administration to mice did not cause skin inflammation, a significant decrease in AQP3 expression was noted (Figure 1B). Therefore, we consider that the inflammatory reaction is not involved in the suppressed expression of AQP3 in the skin caused by erlotinib.

We conducted an in vitro study to determine whether the reduction in AQP3 expression is due to a direct effect of erlotinib on epidermal keratinocytes. For this investigation, the human epidermal keratinocyte cell line HaCaT, which expresses AQP3 and EGFR, was used [39,40]. When erlotinib was added to HaCaT cells, the expression level of AQP3 was decreased in a concentration-dependent manner. This effect was unlikely to be attributable to erlotinib-induced cell damage (Figure 4). Thus, we consider that erlotinib decreases AQP3 expression through a direct effect on epidermal keratinocytes. Erlotinib is well absorbed from the gastrointestinal tract, and its bioavailability is approximately 60% [41]. Continued daily administration of 150 mg erlotinib in humans has been reported to result in a minimum blood concentration of approximately 2.7 μM at a steady state [42]. A positive correlation was demonstrated between the severity of erlotinib-induced skin disorders and the blood drug concentration. The blood concentration of erlotinib in patients with skin disorders has been reported to be approximately 2.2 μM [43]. Therefore, we believe that the concentration of erlotinib added to HaCaT cells in this study was appropriate.

The mechanism of the decreased expression of AQP3 caused by erlotinib was examined by focusing on the Ras/MAPK pathway, which is a signaling pathway that is common to both the anticancer mechanism of erlotinib and the expression regulation mechanism of AQP3. When erlotinib was added to HaCaT cells, erlotinib decreased the expression level of p-EGFR in a concentration-dependent manner without changing the level of EGFR expression. Erlotinib also decreased the expression level of p-ERK in a concentration-dependent manner (Figure 5A). Furthermore, the expression levels of both p-EGFRs and p-ERKs were confirmed to be decreased in mouse skin with decreased AQP3 expression after erlotinib administration (Figure 6). These results correlate with the degree of decreased AQP3 expression caused by erlotinib and suggest that erlotinib suppresses the activation of the Ras/MAPK pathway and decreases the expression of AQP3 in epidermal keratinocytes. It has been reported that AQP8 in cancer cells is involved in cell migration via the EGFR pathway [44]. Therefore, it is possible that the EGFR regulates other AQP family, other than AQP3, in the skin.

Gefitinib, an EGFR-TKI that has the same mechanism of action as erlotinib and a high incidence of dry skin as an adverse reaction [45], also decreased the expression of AQP3 (Figure 5B). This result demonstrates the importance of EGFR signaling in the erlotinib-induced decrease in AQP3. In addition, gefitinib-induced dry skin may also be attributable to decreased AQP3 in the skin.

Based on the above results, erlotinib may cause dry skin via the following mechanism. When erlotinib taken orally reaches the skin after being absorbed in the body, it inhibits EGFR in the skin. As a result, the activity of the Ras/MAPK pathway is suppressed, leading to a suppression of AQP3 transcription. Eventually, the expression level of AQP3 decreases and limits water transport from the vascular side to the corneum side, and the skin becomes dry. Therefore, substances that increase AQP3 expression by signaling pathways other than Ras/MAPK or substances that activate the Ras/MAPK pathway in a skin-specific manner would be effective for treatment or preventing erlotinib-induced dry skin.

## 5. Conclusions

Dry skin due to erlotinib may be caused by the decreased expression of AQP3 in the skin, thereby limiting water transport from the vascular side to the corneum side. The decrease in AQP3 may also be attributable to ERK suppression via inhibition of EGFR activity by erlotinib. Therefore, substances that increase AQP3 expression may be effective for erlotinib-induced dry skin.

## Figures and Tables

**Table 1:** Primer sequences for real-time PCR.

Gene	Forward Primer (5′ to 3′)	Reverse Primer (5′ to 3′)
*mIL-6*	CCACCTTTTGACAGTGATGAG	CCTGAAGCTCTTGTTGATGTG
*mTNF-* *α*	AAGCCTGTAGCCCACGTCGTA	GGCACCACTAGTTGGTTGTCTTTG
*mFilaggrin*	AAGGAAATCAGTCTTGCCGT	CTGACCTTCTGAGACACACC
*mLoricrin*	GCCGATGGGCTTAACTTTCT	CAGGATACACCTTGAGCGAC
*mAcer1*	CCGAGTTCTACAATACGTTCA	CATACGGATGCATGAGGAAC
*mAsah1*	CTGTCCTCAACAAGCTGACTG	TCTCAGTACGTCCTCAAGGC
*mSptlc1*	TCCCCTTCCAGAACTGGTTAAA	CCATAGTGCTCGGTGACT
*mSptlc2*	GTCAGGAAATTGGAAACCTGG	AGCTTCCACACCTAAGAACC
*mHyal1*	TTTCTTTGAGCCTGGAGCTA	GTAGTTTCCTTTCGTTGGCT
*mHas2*	CGTGGATTATGTACAGGTGTGT	CCAACACCTCCAACCATAGG
*mCol1a1*	CCCGAGGTATGCTTGATCTG	GGTGATACGTATTCTTCCGGG
*mCol1a2*	TCTCACTCCTGAAGGCTCTA	GTAGTAATCGCTGTTCCACTC
*mAQP3*	AGACAGCCCCTTCAGGATTT	TCCCTTGCCCTGAATATCTG
*m18S rRNA*	GTCTGTGATGCCCTTAGATG	AGCTTATGACCCGCACTTAC
*hAQP3*	AGACAGCCCCTTCAGGATTT	TCCCTTGCCCTGAATATCTG
*hRPL30*	GAAGACGAAAAAGTCGCTGG	GACCAATTTCGCTTTGCCTT

IL-6: interleukin-6, TNF-α: tumor necrosis factor-α, Acer1: alkaline ceramidase 1, Asah1: acid ceramidase 1, Sptlc1: serine palmitoyltransferase 1, Sptlc2: serine palmitoyltransferase 2, Hyal1: hyaluronidase 1, Has2: hyaluronic acid synthase 2, Col1a1: collagen type I α1 chain, Col1a2: collagen type I α2 chain, RPL30: ribosomal protein L30.
